# MRE11 is essential for the long‐term viability of undifferentiated spermatogonia

**DOI:** 10.1111/cpr.13685

**Published:** 2024-06-18

**Authors:** Zhenghui Tang, Zhongyang Liang, Bin Zhang, Xiaohui Xu, Peng Li, Lejun Li, Lin‐Yu Lu, Yidan Liu

**Affiliations:** ^1^ Key Laboratory of Reproductive Genetics (Ministry of Education), Women's Hospital Zhejiang University School of Medicine Hangzhou China; ^2^ Institute of Translational Medicine Zhejiang University School of Medicine Hangzhou China; ^3^ Zhejiang Provincial Key Laboratory of Precision Diagnosis and Therapy for Major Gynecological Diseases, Women's Hospital Zhejiang University School of Medicine Hangzhou China; ^4^ Zhejiang Key Laboratory of Maternal and Infant Health, Women's Hospital Zhejiang University School of Medicine Hangzhou China; ^5^ Department of Reproductive Endocrinology, Women's Hospital Zhejiang University School of Medicine Hangzhou China; ^6^ Zhejiang University Cancer Center Hangzhou China

## Abstract

In the meiotic prophase, programmed SPO11‐linked DNA double‐strand breaks (DSBs) are repaired by homologous recombination (HR). The MRE11‐RAD50‐NBS1 (MRN) complex is essential for initiating DNA end resection, the first step of HR. However, residual DNA end resection still occurs in *Nbs1* knockout (KO) spermatocytes for unknown reasons. Here, we show that DNA end resection is completely abolished in *Mre11* KO spermatocytes. In addition, *Mre11* KO, but not *Nbs1* KO, undifferentiated spermatogonia are rapidly exhausted due to DSB accumulation, proliferation defects, and elevated apoptosis. Cellular studies reveal that a small amount of MRE11 retained in the nucleus of *Nbs1* KO cells likely underlies the differences between *Mre11* and *Nbs1* KO cells. Taken together, our study not only demonstrates an irreplaceable role of the MRE11 in DNA end resection at SPO11‐linked DSBs but also unveils a unique function of MRE11 in maintaining the long‐term viability of undifferentiated spermatogonia.

## Graphical abstract

The MRE11‐RAD50‐NBS1 (MRN) complex in the nucleus initiates DNA end resection at double‐strand break (DSB) sites and supports the long‐term viability of undifferentiated spermatogonia in mice. In the absence of NBS1, a small amount of intact MRE11‐RAD50 complex can still localise to the nucleus and can promote residual DNA end resection to keep the long‐term viability of undifferentiated spermatogonia. However, in the absence of MRE11, DNA end resection is completely abolished and undifferentiated spermatogonia are rapid exhausted due to DSB accumulation and apoptosis.

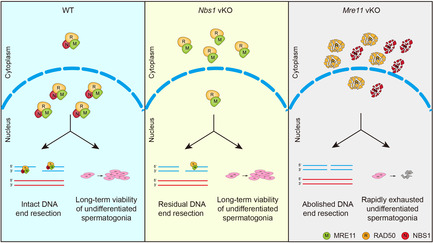

## 1 INTRODUCTION

As one of the most deleterious forms of DNA damage, DNA double‐strand breaks (DSBs) must be repaired promptly to maintain genomic stability. Among multiple pathways of DSB repair, homologous recombination (HR) repairs DSBs with high fidelity when sister chromatids are available as templates in the S and G2 phases of the cell cycle.^1^, ^2^ HR starts with DNA end resection at DSB sites to generate single‐stranded DNA, which is bound by recombinases to initiate the invasion of the DNA template strand and to promote subsequent steps of HR.^3^

In somatic cells, the MRE11‐RAD50‐NBS1 (MRN) complex initiates DNA end resection at DSB sites. As the catalytic subunit of the MRN complex, MRE11 harbours both endonuclease and exonuclease activity.^4^ MRE11 first uses its endonuclease activity to nick the DNA several hundred base pairs away from the DSB site and then uses its 3′‐5′ exonuclease to digest DNA towards the DSB site, generating short single‐stranded DNA. This creates the entry sites for EXO1 and BLM/DNA2, which continue to perform the long‐range resection to generate longer single‐stranded DNA.^5^ Due to the unique dual nuclease activities of MRE11, the MRN complex is essential for DNA end resection at DSBs with blocked ends, such as TOP2‐linked DSBs.^6^

Hundreds of DSBs with blocked ends are generated in the meiotic prophase. After programmed DNA cutting, SPO11 is covalently linked to the 5′ end of DSBs to form SPO11‐linked DSBs, which are repaired exclusively by HR to facilitate the obligate crossover between homologous chromosomes.^7^ The MRX complex, an orthologue of MRN complex in yeast, is essential for DNA end resection in yeast meiotic prophase.^8^ The MRN complex is also required for DNA end resection and HR repair of SPO11‐linked DSBs in mammalian meiotic prophase. Loss of NBS1, a key component of the MRN complex, in spermatocytes results in meiotic arrest and male infertility.^9^

Interestingly, residual DNA end resection still occurs in *Nbs1* knockout (KO) spermatocytes, raising the possibility that other proteins can function as backup nucleases to initiate DNA end resections at SPO11‐linked DSBs when the MRN complex is absent. However, although EXO1 and BLM/DNA2 can initiate DNA end resection at DSBs with clean ends, no proteins are known to have dual nuclease activities similar to those of MRE11, which are essential for DNA end resection at DSBs with blocked ends.^10^ Alternatively, MRE11 is still responsible for the residual DNA end resection in *Nbs1* KO spermatocytes. As NBS1 loss does not affect the stability of the MRE11‐RAD50 complex but compromises its localization at DSBs,^11^, ^12^ it is possible that a small amount of MRE11‐RAD50 complex still localises at DSBs and promotes limited DNA end resection in the absence of NBS1. These two possibilities can be tested by inactivating MRE11 in spermatocytes.

Like *Nbs1* KO mice, both *Mre11* KO and *Mre11* nuclease‐dead mice are lethal, preventing direct examination of meiotic prophase in these mice. Two viable *Mre11* mutant mice are also generated, but none of them have defects in DNA end resection or HR repair in the meiotic prophase.^13^, ^14^, ^15^ In this study, we have generated germ cell‐specific *Mre11* KO mice and found that MRE11 is absolutely required for DNA end resection in meiotic prophase. In addition, we have discovered that *Mre11* KO, but not *Nbs1* KO, leads to complete loss of germ cells due to apoptosis in undifferentiated spermatogonia. Therefore, we have identified a unique role of MRE11 in maintaining the long‐term viability of undifferentiated spermatogonia.

## 2 RESULTS

### 2.1 MRE11 is required for male fertility in mice

**FIGURE 1:**
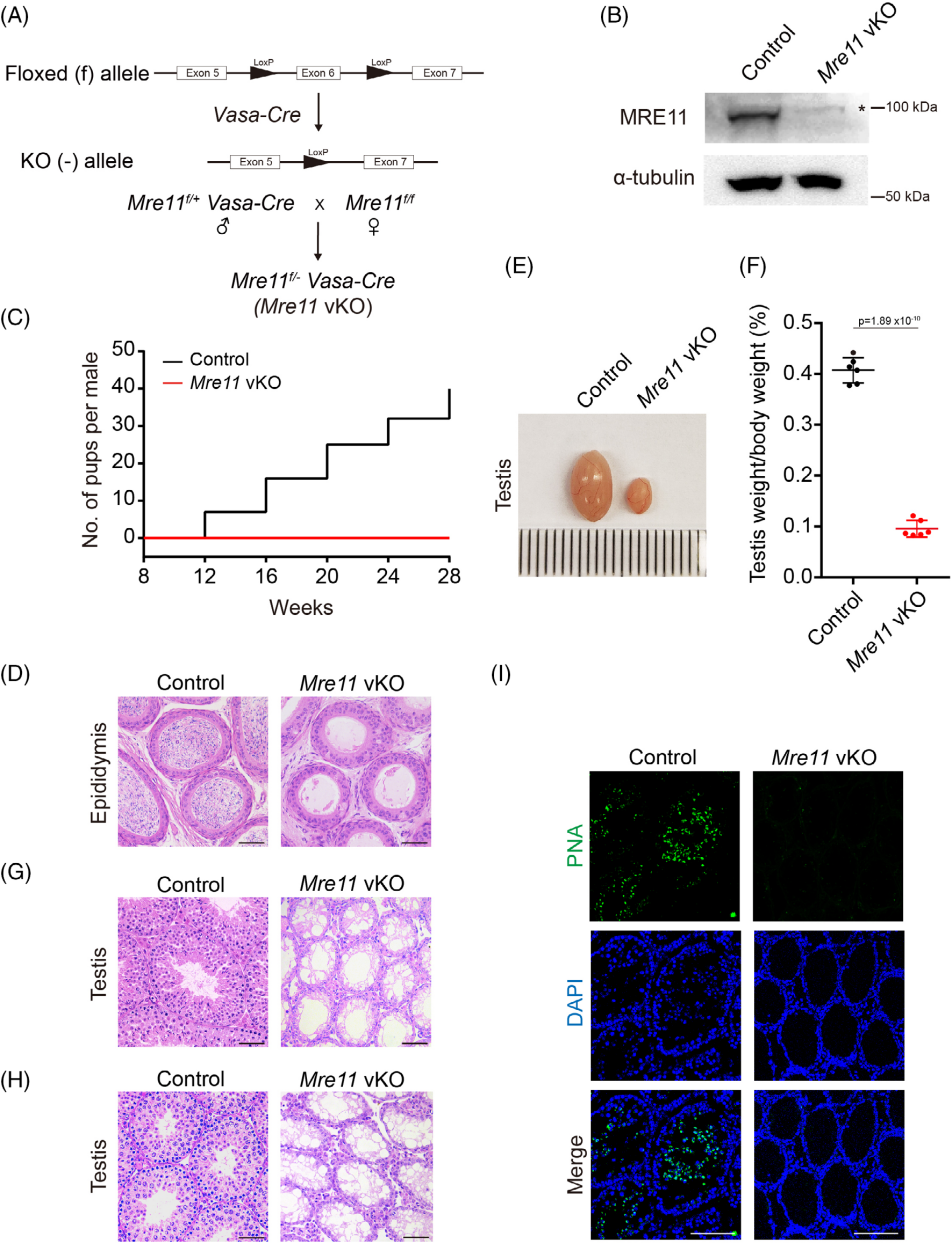
MRE11 is required for male fertility in mice. (A) Schematic illustration of mating strategies to obtain *Mre11* vKO mice. (B) Western blotting analysis of MRE11 protein level in spermatocyte of *Mre11* vKO mice at postnatal day 21 (PD21). α‐tubulin was used as loading control. Asterisk marks nonspecific band. (C) Cumulative numbers of pups per male obtained by mating Control or *Mre11* vKO male mice with WT female mice. Three breeding pairs were used. (D) Haematoxylin and eosin (H/E) staining of paraffin sections from Control and *Mre11* vKO epididymides at PD42. (E) Testis size comparison of Control and *Mre11* vKO male mice at PD21. Six testes were measured. (F) Ratios of testis weight to body weight of Control and *Mre11* vKO male mice at PD42. (G, H) H/E staining of paraffin sections of testes from Control and *Mre11* vKO male mice at PD42 (G) and PD21 (H). (I) PNA staining of testes frozen sections from Control and *Mre11* vKO mice at PD21. Scale bars, 100 μm.

To investigate if MRE11 is essential for DNA end resection and HR repair of SPO11‐linked DSBs in mammalian meiotic prophase, we generated germ cell‐specific *Mre11* KO (*Mre11* vKO) mice using *Vasa‐Cre* (Figure 1A). Successful depletion of MRE11 protein in *Mre11* vKO testes was verified by western blotting (Figure 1B). Similar to *Nbs1* vKO male mice that we generated previously, *Mre11* vKO male mice were infertile (Figure 1C). No mature sperm was found in the epididymides of adult *Mre11* vKO male mice (Figure 1D). The testes of *Mre11* vKO male mice were smaller (Figure 1E,F). In the testis sections of *Mre11* vKO male mice, no haploid cells were found by H/E staining (Figure 1G,H), and no cells with acrosomes were identified by peanut agglutinin (PNA) staining (Figure 1I). These observations were identical to those in *Nbs1* vKO male mice, suggesting that both MRE11 and NBS1 are essential for the completion of meiosis in male mice.

### 2.2 MRE11 is required for DNA end resection at SPO11‐linked DSBs

**FIGURE 2:**
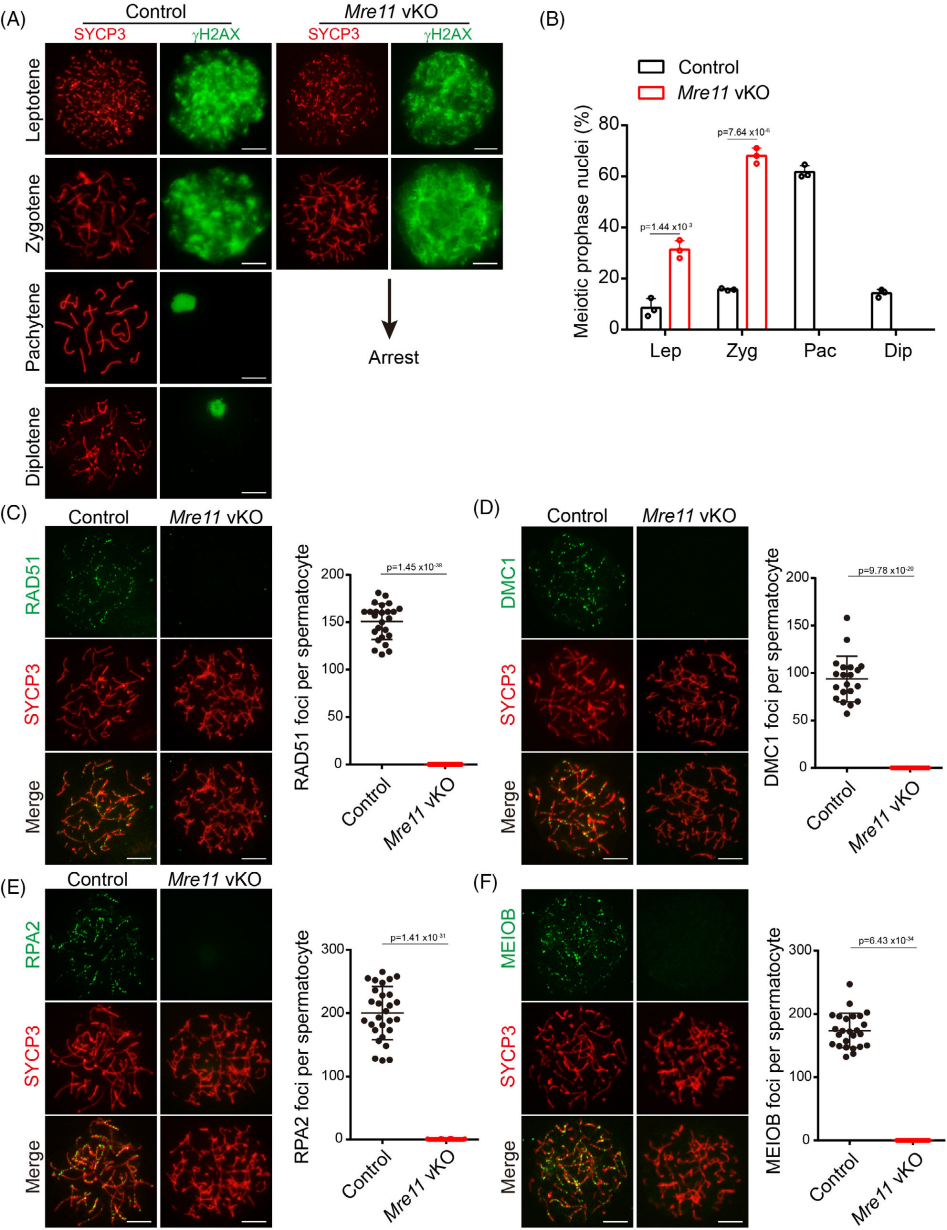
MRE11 is required for DNA end resection at SPO11‐linked DSBs in meiosis. (A). Immunofluorescent (IF) staining of SYCP3 and γH2AX to distinguish different stages of meiotic prophase in surface spread of spermatocytes from Control and *Mre11* vKO male mice at PD21. Scale bars, 10 μm. (B). Percentage of spermatocytes at each stage of meiotic prophase (Lep, leptotene; Zyg, zygotene; Pac, pachytene; Dip, diplotene). Error bars represent SEM. Three mice per genotype and 100 cells in each mouse were analysed. (C–F) Representative images of RAD51 (C), DMC1 (D), RPA2 (E), and MEIOB (F) IF staining in the zygotene‐stage spermatocytes from Control and *Mre11* vKO male mice at PD21. The statistical charts of RAD51, DMC1, RPA2, and MEIOB foci number between the two groups were shown on the right. Error bars represent SEM, and 50 cells in each group were analysed. Scale bars, 10 μm.

To dissect the meiotic defects in *Mre11* vKO male mice, we first analysed the meiotic prophase in these mice (Figure 2A). The intensity of γH2AX signals in leptotene‐stage spermatocytes of *Mre11* vKO mice was indistinguishable from that in control mice, suggesting that there is no defect in the formation of programed SPO11‐linked DSBs in *Mre11* vKO mice. However, diminished γH2AX signals could not be observed in any spermatocytes at meiotic prophase, suggesting that DSB repair was severely compromised in *Mre11* vKO male mice. Concomitantly, meiotic progression was arrested at the zygotene stage in these mice (Figure 2B). The HR repair of SPO11‐linked DSBs in the meiotic prophase requires two meiotic recombinases, RAD51 and DMC1. In the zygotene‐stage spermatocytes of *Mre11* vKO male mice, both recombinases were undetectable at DSB sites (Figure 2C,D). The recruitment of recombinases to DSBs is mediated by single‐stranded DNA, a product of DNA end resection. The single‐stranded binding proteins, RPA2 and MEIOB, were also undetectable at DSB sites (Figure 2E,F). These observations suggest that DNA end resection is dramatically decreased below our detection limit in the meiotic prophase in *Mre11* vKO male mice, which is the reason for DSB repair defects in these mice. Notably, unlike *Nbs1* vKO spermatocytes, no residual DNA end resection could be observed in *Mre11* vKO spermatocytes. Therefore, MRE11 is likely the only nuclease that initiates DNA end resection at SPO11‐linked DSBs in meiotic prophase, which is responsible for the residual DNA end resection in *Nbs1* KO spermatocytes.

### 2.3 MRE11 is essential for the long‐term viability of undifferentiated spermatogonia

**FIGURE 3:**
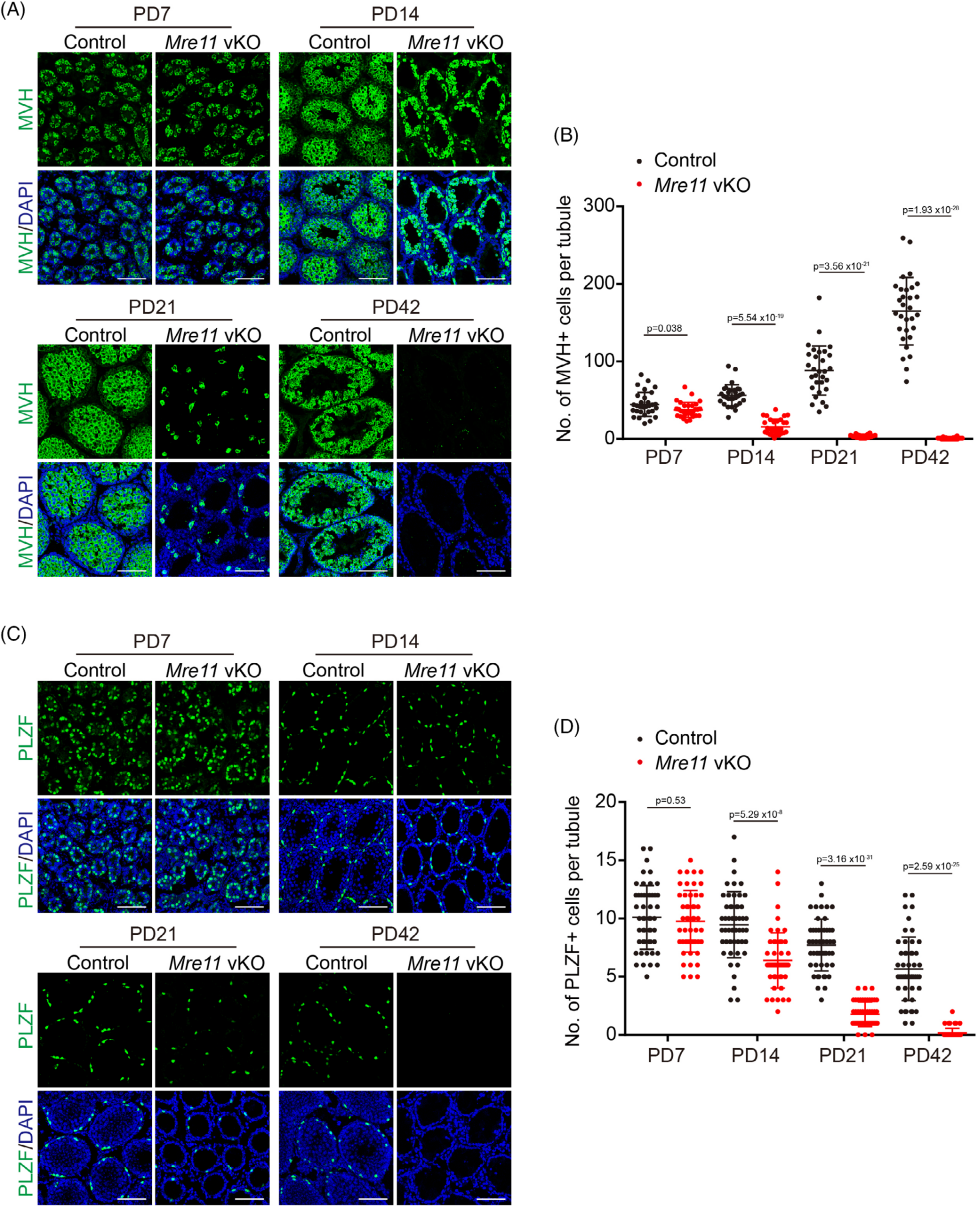
MRE11 is essential for the long‐term viability of undifferentiated spermatogonia. (A) IF staining of MVH in testes frozen sections from Control and *Mre11* vKO mice at PD7, PD14, PD21, and PD42. (B) Quantification of MVH+ cells per seminiferous tubule from Control and *Mre11* vKO mice. (C). IF staining of PLZF in testes frozen sections from Control and *Mre11* vKO mice at PD7, PD14, PD21, and PD42. (D). Quantification of PLZF+ cells per seminiferous tubule from Control and *Mre11* vKO mice. Error bars represent SEM, and 30 tubules in each group were analysed. Scale bars, 80 μm.

During the analysis of DNA end resection, we noticed that much fewer spermatocytes at meiotic prophase could be obtained from the testes of *Mre11* vKO male mice than from control mice at postnatal day (PD) 21. Examination of testis sections of *Mre11* vKO male mice after H/E staining also revealed a dramatic reduction of spermatocytes at meiotic prophase at PD21 (Figure 1H). These observations suggest that *Mre11* vKO male mice have additional defects in germ cells besides meiotic prophase arrest in spermatocytes. To characterise the germ cell defects, we analysed the germ cells in control and *Mre11* vKO male mice of different ages by immunofluorescent (IF) staining of MVH (Figure 3A), which is expressed in germ cells at all stages from primordial germ cells to round spermatids.^16^ The numbers of germ cells were comparable between control and *Mre11* vKO male mice at PD7 but were decreased in *Mre11* vKO male mice at PD14. Consistent with our initial findings, a dramatic reduction of germ cells in *Mre11* vKO male mice was observed at PD21. Three weeks later, germ cells could be barely observed in *Mre11* vKO male mice at PD42 (Figure 3A,B).

The near complete absence of germ cells at PD42 indicates that the undifferentiated spermatogonia, which maintain long‐term spermatogenesis, are rapidly exhausted in *Mre11* vKO male mice. Therefore, we analysed the undifferentiated spermatogonia in control and *Mre11* vKO male mice of different ages by IF staining of PLZF (Figure 3C), a marker of undifferentiated spermatogonia. The undifferentiated spermatogonia were similar between control and *Mre11* vKO male mice at PD7, suggesting that the establishment of undifferentiated spermatogonia was normal. The decrease of undifferentiated spermatogonia in *Mre11* vKO male mice was first observed at PD14 and was more dramatic at PD21. The undifferentiated spermatogonia were almost undetectable in *Mre11* vKO male mice at PD42 (Figure 3C,D). These observations suggest that MRE11 is essential for the long‐term viability of undifferentiated spermatogonia.

### 2.4 NBS1 is dispensable for the long‐term viability of undifferentiated spermatogonia

**FIGURE 4:**
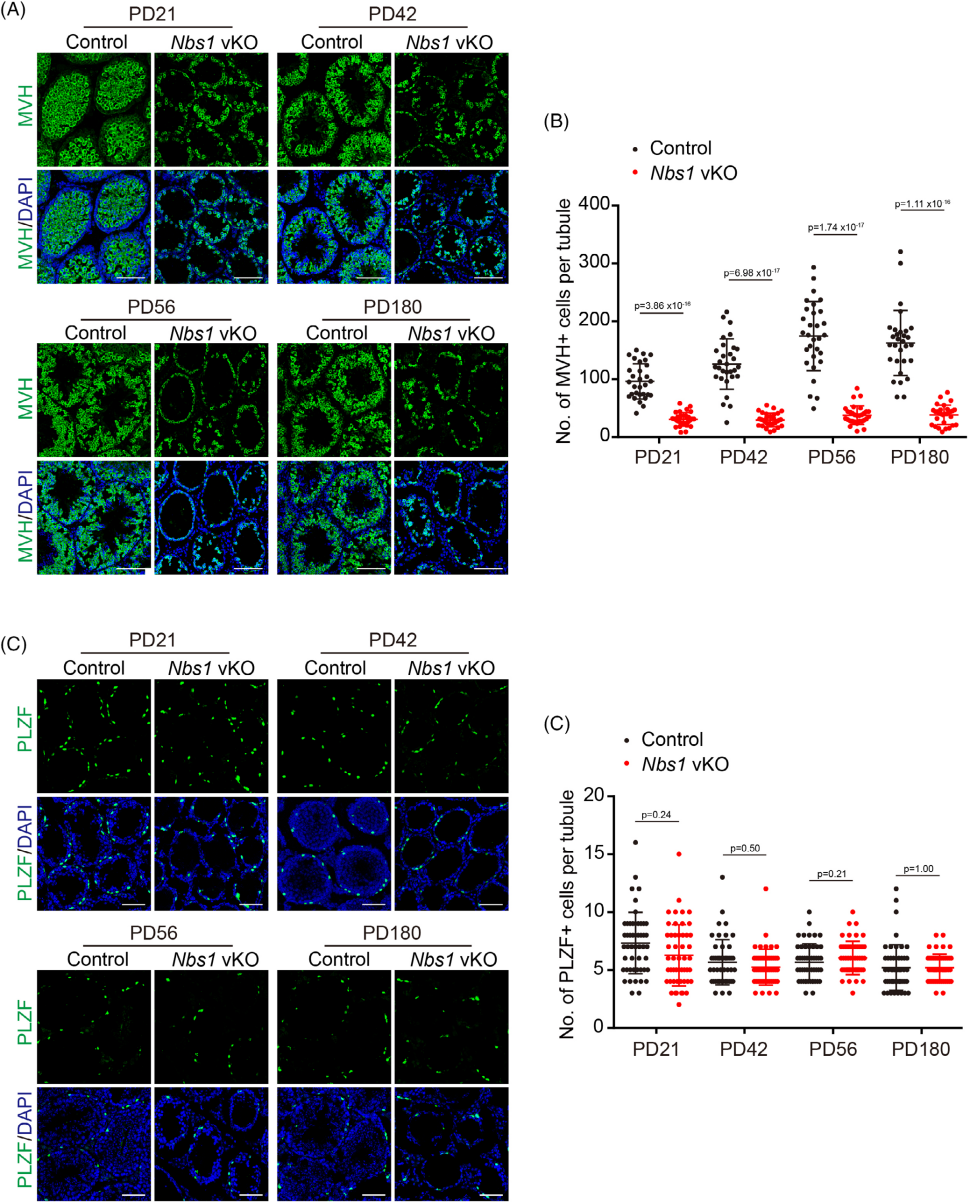
NBS1 is dispensable for the long‐term viability of undifferentiated spermatogonia. (A) IF staining of MVH in testes frozen sections from Control and *Nbs1* vKO mice at PD21, PD42, PD56, and PD180. (B) Quantification of MVH+ cells per seminiferous tubule from Control and *Nbs1* vKO mice. (C) IF staining of PLZF in testes frozen sections from Control and *Nbs1* vKO mice at PD21, PD42, PD56, and PD180. (D) Quantification of PLZF+ cells per seminiferous tubule from Control and *Nbs1* vKO mice. Error bars represent SEM, and 30 tubules in each group were analysed. Scale bars, 80 μm.

In most scenarios, MRE11 and NBS1 function together as an MRN complex, and their deficiency leads to similar phenotypes in most studies. Although *Mre11* vKO and *Nbs1* vKO male mice have subtle differences in DNA end resection defects, the meiotic prophase defects are similar. However, unlike *Mre11* vKO male mice, our previous study did not notice a dramatic reduction of undifferentiated spermatogonia in *Nbs1* vKO male mice. In case any defects in *Nbs1* vKO male mice were missed previously, we revisited whether NBS1 is required for the long‐term viability of undifferentiated spermatogonia. Like *Mre11* vKO male mice, germ cells were dramatically reduced in *Nbs1* vKO male mice at PD21. However, unlike *Mre11* vKO male mice, germ cells were still abundant at PD42 and were present at PD180 in *Nbs1* vKO male mice (Figure 4A,B). Interestingly, the numbers of undifferentiated spermatogonia were similar between control and *Nbs1* vKO male mice at all ages analysed, including PD180, suggesting that undifferentiated spermatogonia were not exhausted in *Nbs1* vKO male mice (Figure 4C,D). The reduction of germ cells in *Nbs1* vKO male mice is likely due to meiotic prophase arrest only. These observations suggest that, unlike MRE11, NBS1 is dispensable for the long‐term viability of undifferentiated spermatogonia.

### 2.5 MRE11 loss leads to DSB accumulation and apoptosis in undifferentiated spermatogonia

**FIGURE 5:**
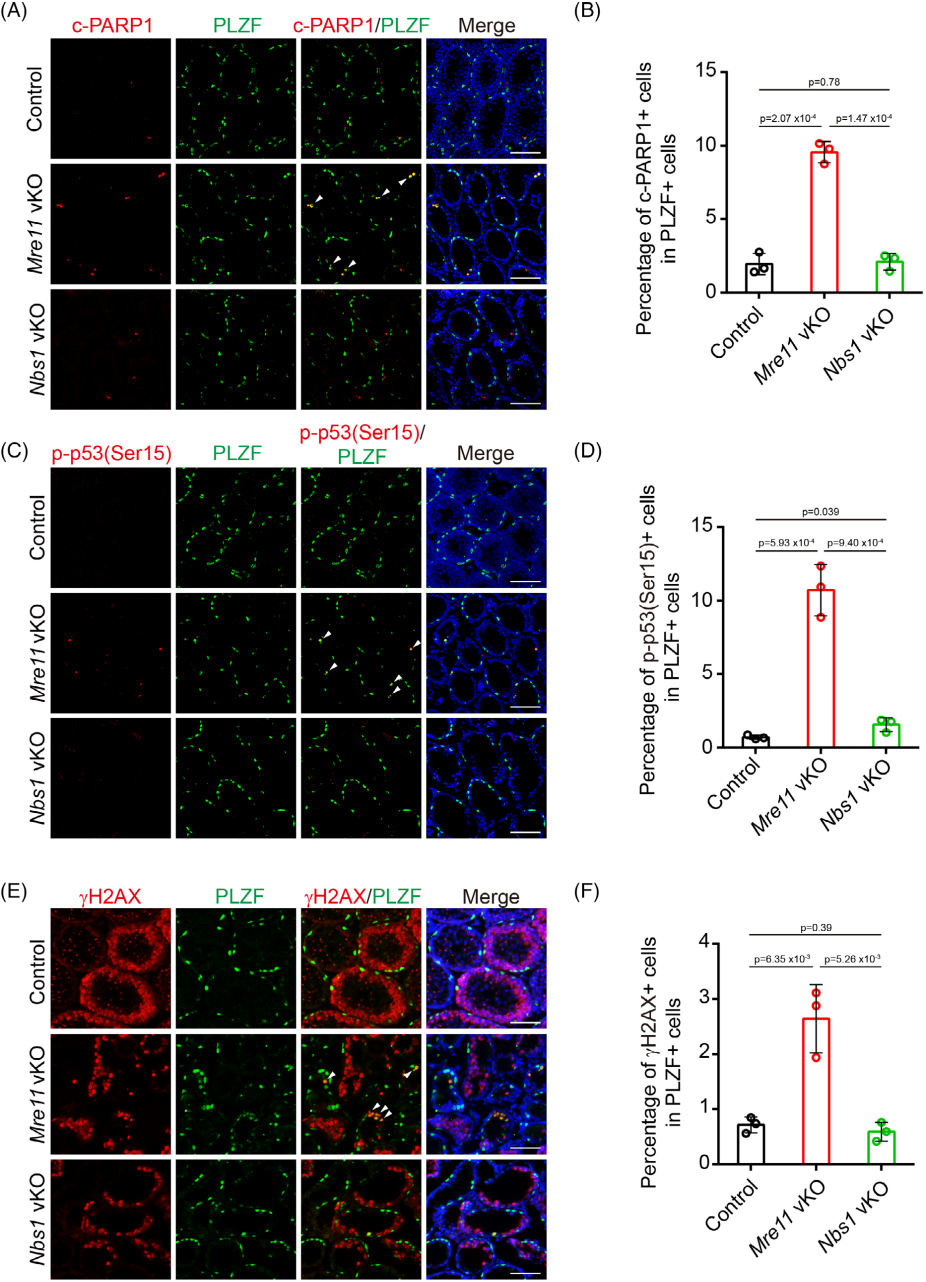
MRE11 loss leads to DSB accumulation and apoptosis in undifferentiated spermatogonia. (A) IF staining of cleaved‐PARP1 (c‐PARP1) and PLZF in testes frozen sections from Control, *Mre11* vKO, and *Nbs1* vKO mice at PD14. Arrows indicate c‐PARP1 and PLZF double‐positive cells. (B) Statistical analysis of c‐PARP1+ cell in PLZF+ cells per seminiferous tubule from Control, *Mre11* vKO, and *Nbs1* vKO mice. (C) IF staining of p‐p53(Ser15) and PLZF in frozen sections of testes from Control, *Mre11* vKO, and *Nbs1* vKO mice at PD14. Arrows indicate the p‐p53(Ser15) and PLZF double‐positive cells. (D) Statistical analysis of p‐p53(Ser15) + cell in PLZF+ cells per seminiferous tubule from Control, *Mre11* vKO, and *Nbs1* vKO mice. (E) IF staining of γH2AX and PLZF in frozen sections of testes from Control, *Mre11* vKO, and *Nbs1* vKO mice at PD14. Arrows indicate the γH2AX and PLZF double‐positive cells. (F) Statistical analysis of γH2AX+ cell in PLZF+ cells per seminiferous tubule from Control, *Mre11* vKO, and *Nbs1* vKO mice. Error bars represent SEM. Three mice per genotype and 200 cells in each mouse were analysed. Scale bars, 80 μm.

To investigate why undifferentiated spermatogonia are exhausted in *Mre11* vKO but not *Nbs1* vKO male mice, we examined undifferentiated spermatogonia in these mice at PD14, when the decrease of undifferentiated spermatogonia was first observed in *Mre11* vKO male mice. Compared with control mice, the number of undifferentiated spermatogonia positive for cleaved PARP1 was significantly increased in *Mre11* vKO male mice (Figure 5A,B). The signal of p‐p53(S15), a phosphorylation event associated with p53 activation, was also dramatically increased in *Mre11* KO undifferentiated spermatogonia. These observations suggest that many *Mre11* KO undifferentiated spermatogonia are undergoing apoptosis at PD14 (Figure 5C,D). p53 can be robustly activated by DNA damage.^17^ As MRE11 is essential for DSB repair, the elevated apoptosis in *Mre11* KO undifferentiated spermatogonia may be caused by DSB repair deficiency. To test this idea, we examined the status of γH2AX, a marker of DSBs, in *Mre11* vKO male mice. In control mice, most γH2AX signals were found in spermatocytes when programmed DSBs were generated in meiotic prophase, and γH2AX signals could hardly be identified in undifferentiated spermatogonia (Figure 5E). On the contrary, the number of undifferentiated spermatogonia positive for γH2AX was significantly increased in *Mre11* vKO male mice (Figure 5E,F). Therefore, DSBs were indeed present in *Mre11* KO undifferentiated spermatogonia, which could be the cause for the elevated apoptosis in these cells. In sharp contrast to the observations in *Mre11* vKO male mice, the number of undifferentiated spermatogonia positive for cleaved PARP1, p‐p53(S15), or γH2AX in *Nbs1* vKO male mice was comparable with those in control mice (Figure 5). The different levels of persistent DSBs likely account for the different fates of *Mre11* KO and *Nbs1* KO undifferentiated spermatogonia.

### 2.6 MRE11 is required for the proliferation of undifferentiated spermatogonia

**FIGURE 6:**
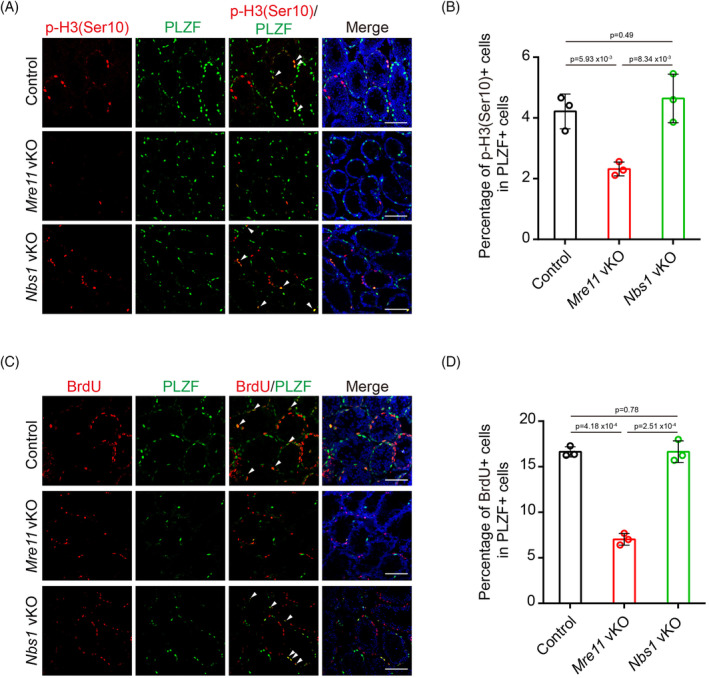
MRE11 is required for the proliferation of undifferentiated spermatogonia. (A) IF staining of p‐H3(Ser10) and PLZF in testes frozen sections from Control, *Mre11* vKO, and *Nbs1* vKO mice at PD14. Arrows indicate the p‐H3(Ser10) and PLZF double‐positive cells. (B) Statistical analysis of p‐H3(Ser10) + cell in PLZF+ cells per seminiferous tubule from Control, *Mre11* vKO, and *Nbs1* vKO mice. Error bars represent SEM. Three mice per genotype were analysed. (C) IF staining of BrdU and PLZF in testes frozen sections from Control, *Mre11* vKO, and *Nbs1* vKO mice at PD14. Arrows indicate the BrdU and PLZF double‐positive cells. (D) Statistical analysis of BrdU+ cell in PLZF+ cells per seminiferous tubule from Control, *Mre11* vKO, and *Nbs1* vKO mice. Error bars represent SEM. Three mice per genotype and 200 cells in each mouse were analysed. Scale bars, 80 μm.

Besides triggering apoptosis, persistent DSBs can also activate cell cycle checkpoints to cease cell proliferation. In agreement with this idea, the signal of p‐H3(S10), a phosphorylation event associated with mitotic entry, was significantly decreased in *Mre11* KO undifferentiated spermatogonia (Figure 6A,B). On the contrary, p‐H3(S10) signals were comparable between control and *Nbs1* KO undifferentiated spermatogonia. Similarly, the percentage of cells undergoing DNA replication, as determined by BrdU incorporation, was significantly decreased in *Mre11* KO but not in *Nbs1* KO undifferentiated spermatogonia (Figure 6C,D). Therefore, proliferation defects were present in *Mre11* KO but not in *Nbs1* KO undifferentiated spermatogonia. It is likely that the proliferation defects and apoptosis in *Mre11* KO undifferentiated spermatogonia eventually lead to the exhaustion of these cells in older mice.

### 2.7 NBS1 loss compromises but not fully abolishes the nuclear localization of MRE11‐RAD50 complex

**FIGURE 7:**
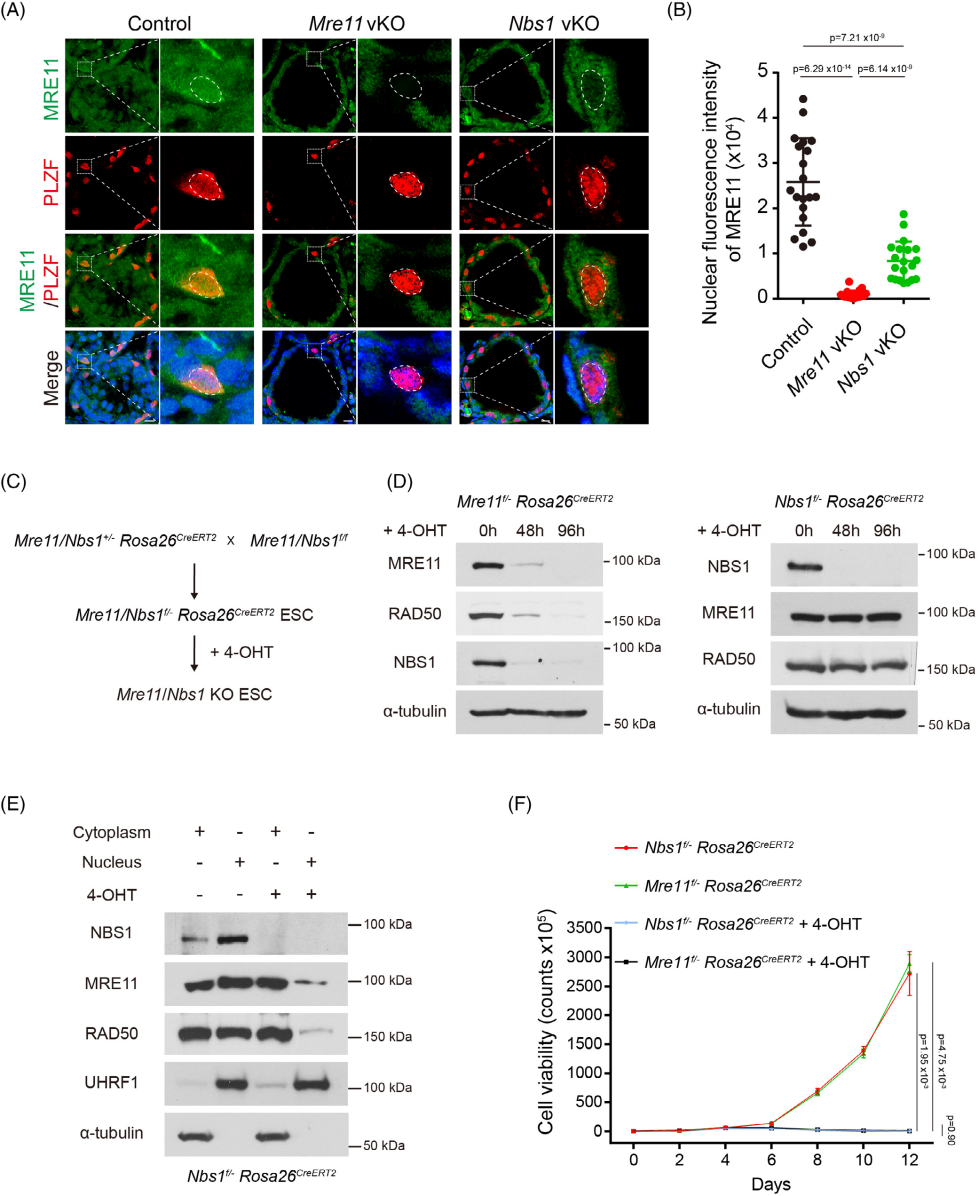
NBS1 loss compromises but not fully abolishes the nuclear localization of MRE11‐RAD50 complex. (A) IF staining of MRE11 and PLZF in testes frozen sections from Control, *Mre11* vKO, and *Nbs1* vKO mice at PD14. Scale bars, 20 μm. (B) Quantification of nuclear fluorescence intensity of MRE11 in undifferentiated spermatogonia of Control, *Mre11* vKO, and *Nbs1* vKO mice. 20 cells in each group were analysed. (C) Strategies to generate inducible *Mre11* KO (*Mre11* ^*f/−*^ *Rosa26* ^*CreERT2*^) and *Nbs1* KO (*Nbs1* ^*f/−*^ *Rosa26* ^*CreERT2*^) ESCs. (D) Western blotting analysis of the protein levels of MRE11, NBS1, and RAD50 in *Nbs1* ^*f/−*^ *Rosa26* ^*CreERT2*^ and *Mre11* ^*f/−*^ *Rosa26* ^*CreERT2*^ ESCs after 4‐hydroxytamoxifen (4‐OHT) treatment. α‐tubulin was used as loading control. (E) Western blotting analysis of cytoplasmic and nuclear distribution of MRE11 and RAD50 in *Nbs1* ^*f/−*^ *Rosa26* ^*CreERT2*^ ESCs after 4‐OHT treatment. α‐tubulin and UHRF1 were used as loading controls for cytoplasm and nucleus, respectively. (F) Cell viability assay of *Nbs1* ^*f/−*^ *Rosa26* ^*CreERT2*^ and *Mre11* ^*f/−*^ *Rosa26* ^*CreERT2*^ ESCs with and without 4‐OHT treatment.

Previous studies have shown that NBS1 is dispensable for the stability of the MRE11‐RAD50 complex, but it is required for the localization of the MRE11‐RAD50 complex in the nucleus. Interestingly, unlike the absence of MRE11 signals in the nucleus of *Mre11* KO undifferentiated spermatogonia, weak MRE11 signals could still be detected in the nucleus of *Nbs1* KO undifferentiated spermatogonia (Figure 7A,B). Although the status of RAD50 could not be directly analysed due to the unavailability of RAD50 antibody for IF staining, it is likely that NBS1 loss compromises but not fully abolishes the nuclear localization of MRE11‐RAD50 complex. It is possible that complete loss of MRE11 may be detrimental to cell proliferation and viability of *Mre11* KO undifferentiated spermatogonia, but a small amount of intact MRE11‐RAD50 complex in the nucleus is sufficient for the proliferation and viability of *Nbs1* KO undifferentiated spermatogonia.

Multiple studies have shown that both MRE11 and NBS1 are essential for cell proliferation and long‐term cell viability. Interestingly, our study reveals that MRE11, but not NBS1, is required for cell proliferation and the long‐term viability of undifferentiated spermatogonia in vivo. Therefore, we continued to examine if loss of MRE11 and NBS1 have different impact on the proliferation of cells cultured in vitro. As undifferentiated spermatogonia are challenging to culture in vitro, we utilised embryonic stem cells (ESCs). We generated inducible *Mre11* KO (*Mre11* ^*f/−*^ *Rosa26* ^*CreERT2*^) and inducible *Nbs1* KO (*Nbs1* ^*f/−*^ *Rosa26* ^*CreERT2*^) ESCs, which proliferated well in vitro (Figure 7C). Upon 4‐hydroxytamoxifen (4‐OHT) treatment, complete *Mre11* KO and *Nbs1* KO ESCs were obtained within 4 days (Figure 7D). In agreement with previous studies, the protein levels of RAD50 and NBS1 were dramatically decreased in *Mre11* KO ESCs, but the protein levels of both MRE11 and RAD50 were unaltered in *Nbs1* KO ESCs (Figure 7D). Consistent with our observation in *Nbs1* KO undifferentiated spermatogonia and a previous study in Purkinje cells,^18^ the nuclear localization of MRE11 and RAD50 were severely compromised but still detectable in *Nbs1* KO ESCs (Figure 7E). Interestingly, unlike undifferentiated spermatogonia in vivo, both *Mre11* KO and *Nbs1* KO ESCs were deficient in proliferation after 4 days and died eventually (Figure 7F), suggesting that the amount of MRE11‐RAD50 complex is not sufficient for supporting the proliferation and viability of *Nbs1* KO ESCs in vitro. Further investigation is required to uncover the mechanism underlying this observation.

## 3 DISCUSSION

MRE11 is the catalytic subunit of the MRN complex that initiates DNA end resection at DSBs. Besides the MRN complex, other nucleases can also initiate DNA end resection at DSBs with clean ends.^19^, ^20^, ^21^ In this study, we have demonstrated that DNA end resection is dramatically decreased below our detection limit in *Mre11* KO spermatocytes, suggesting that other nucleases cannot substitute the MRN complex to initiate DNA end resection at SPO11‐linked DSBs. Most nucleases promote DNA end resection in a 5′‐3′ direction, requiring a free 5′ end to initiate.^22^, ^23^ However, the 5′ ends of all DSBs in the meiotic prophase are covalently linked with SPO11, which blocks the access of these nucleases.^24^ This unique endonuclease and 3′‐5′ exonuclease activities of MRE11 enable the MRN complex to nick the DNA several hundred base pairs away from DSBs and digest the DNA in a 3′‐5′ direction towards SPO11‐linked DSBs to remove SPO11 from DSBs, creating the entry sites for other nuclease to continue the DNA end resection in a 5′‐3′ direction.^5^ No enzyme with similar activities as MRE11 has ever been identified, and our study suggests that such an enzyme does not exist, at least in the meiotic prophase.

**FIGURE 8:**
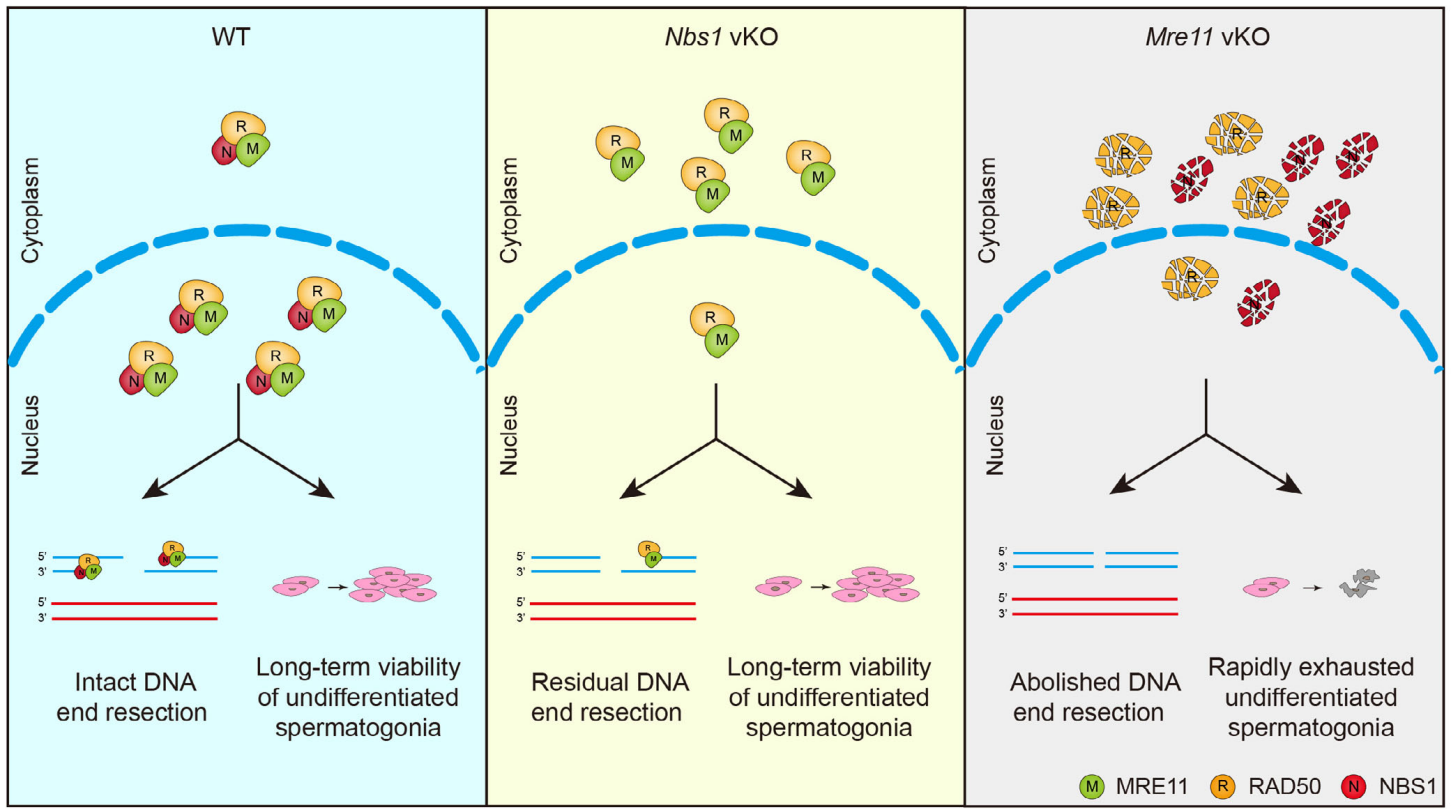
Working model: MRE11 is essential for DNA end resection and the long‐term viability of undifferentiated spermatogonia. (A) In wild‐type (WT) mice, the MRN complex in the nucleus initiates DNA end resection at DSB sites and supports the long‐term viability of undifferentiated spermatogonia. (B) In *Nbs1* vKO mice, a small amount intact MRE11‐RAD50 complex localises to the nucleus and promotes residual DNA end resection, which is sufficient for the long‐term viability of undifferentiated spermatogonia. (C) In *Mre11* vKO mice, DNA end resection is completely abolished and undifferentiated spermatogonia are rapid exhausted due to DSB accumulation and apoptosis.

As a critical component of the MRN complex, NBS1 is required for DNA end resection at SPO11‐linked DSBs in meiotic prophase, but residual DNA end resection still occurs in *Nbs1* KO spermatocytes. Our study using *Mre11* vKO male mice suggests that MRE11, but not other nucleases, is responsible for the residual DNA end resection when NBS1 is lost. In *Nbs1* KO cells, the MRE11‐RAD50 complex is intact, and a small amount of it can be observed in the nucleus, which is likely functional to promote residual DNA end resection in the absence of NBS1. However, such residual DNA end resection is insufficient for meiotic recombination at all DSBs. Therefore, the meiotic prophase progression arrests at similar stages in *Mre11* KO and *Nbs1* KO spermatocytes (Figure 8).

Unlike in meiotic prophase, loss of MRE11 and NBS1 leads to dramatically different outcomes in undifferentiated spermatogonia. While *Nbs1* KO undifferentiated spermatogonia are indistinguishable from those in WT mice, *Mre11* KO undifferentiated spermatogonia are rapidly exhausted. Before exhaustion, *Mre11* KO undifferentiated spermatogonia display defects in cell proliferation, accompanied by the accumulation of unrepaired DSBs and elevated apoptosis. Therefore, MRE11, but not NBS1, is required for cell proliferation and long‐term viability of undifferentiated spermatogonia (Figure 8). As *Mre11* vKO male mice have no defects in establishing undifferentiated spermatogonia at PD7, it is likely that MRE11 is dispensable for DNA replication per se. However, the MRN complex has an active role in fixing endogenous replication errors by repairing collapsed replication forks and resolving replication intermediates, the defect of which leads to DSB accumulation.^25^, ^26^ In the absence of replication stress induced by exogenous DNA damage, replication errors occur infrequently in vivo. A small amount of intact MRE11‐RAD50 complex in *Nbs1* KO undifferentiated spermatogonia may be sufficient for fixing these replication errors. On the contrary, replication errors cannot be fixed in *Mre11* KO undifferentiated spermatogonia, and DSBs gradually accumulate, eventually leading to proliferation arrest, apoptosis, and cell exhaustion. While weak MRE11 signals can be observed in *Nbs1* KO undifferentiated spermatogonia, the status of RAD50 could not be analysed due to technical constraint of RAD50 antibodies. Therefore, this idea needs to be further interrogated using a suitable RAD50 antibody in future.

Our observations in undifferentiated spermatogonia suggest that loss of MRE11 and NBS1 can lead to different outcomes in vivo. Interestingly, loss of MRE11 and NBS1 in Purkinje cells leads to similar phenotypes, both of with are compatible with cerebellar development.^18^ It is possible that the similar or different phenotypes upon loss of MRE11 and NBS1 depend on cell types and their proliferation status in vivo. Consistently, human germline mutations in *MRE11* and *NBS1* genes predispose to A‐T like disease (ATLD) and Nijmegen breakage syndrome (NBS), respectively, which are similar but not identical syndromes.^11^, ^27^, ^28^ Both syndromes are characterised by immunodeficiency and radiosensitivity. While microcephaly is a common feature of NBS but not ATLD, neurodegeneration is more frequently identified in ATLD than in NBS.^29^ In addition, similar but also different phenotypes are observed in mouse models of ATLD and NBS.^13^, ^30^ Therefore, although MRE11, RAD50, and NBS1 functions as a complex, loss or mutation of individual components of this complex can lead to similar or different outcomes in vivo, depending on the cell types and their proliferation status.

Interestingly, *Nbs1* KO ESCs have cell proliferation defects similar to *Mre11* KO ESCs and fail to survive the long‐term culture in vitro, which differs from *Nbs1* KO undifferentiated spermatogonia in vivo. This observation is consistent with the conclusion from previous studies that the loss of either component of the MRN complex is incompatible with cell viability.^31^, ^32^, ^33^ The cell proliferation defects of in vitro cultured *Nbs1* KO ESCs can be explained by the higher replication stress, which can potentially be induced by any environmental factors that are different from those in vivo, such as culture medium, stiffness of the culture plate, monolayer culture, higher oxygen level, etc. It is likely that the small amount of intact MRE11‐RAD50 complex in *Nbs1* KO cells is not sufficient for fixing higher numbers of replication errors that are induced by higher replication stress in vitro.

Both *Mre11* KO and *Nbs1* KO mice die at early embryonic stages,^31^, ^33^ but the reasons are unclear. Based on the cellular studies in vitro, it is believed that loss of either component of the MRN complex is incompatible with cell viability, and both *Mre11* KO and *Nbs1* KO embryos die because of cell proliferation defects.^34^, ^35^ However, our study suggests that the observation in vitro might not fully reflect the situations in vivo. Instead, our study shows that MRE11, but not NBS1, is required for long‐term cell viability of undifferentiated spermatogonia in vivo. It is possible that NBS1 is dispensable for cell proliferation but required for certain critical events during embryonic development. It will be interesting to examine if different mechanisms underlie the embryonic lethality of *Mre11* KO and *Nbs1* KO mice.

## 4 MATERIALS AND METHODS

### 4.1 Mice

*Mre1* ^*f/f*^ mice were kind gifts from David Ferguson (University of Michigan). *Nbs1* ^*f/f*^ mice were kind gifts from Zhao‐Qi Wang (Fritz Lipmann Institute). *Vasa‐Cre* transgenic mice (The Jackson Laboratory, 006954) and *Rosa26* ^*CreERT2*^ mice (The Jackson Laboratory, 008463) were used to generate *Mre11* or *Nbs1* conditional KO mice. All mice experiments were approved by Zhejiang University Animal Care and Use Committee.

### 4.2 Reagents and antibodies

The following reagents and antibodies were purchased: PNA (Sigma, C7381), 4‐OHT (Sigma, H17), BrdU (Sigma, B5002), anti‐MRE11 (CST, 4859S), anti‐MRE11 (NOVUS, NB100‐142), anti‐NBS1 (Abcam, ab32074), anti‐RAD50 (ABclonal, A3078), anti‐UHRF1 (Santa Cruz, sc‐373,750), anti‐α‐tubulin (Genscript, A01410‐100), anti‐γH2AX (Millipore, 05–636), mouse anti‐SYCP3 (Santa Cruz, sc‐74,569), rabbit anti‐SYCP3 (Proteintech, 23,024‐1‐AP), anti‐RPA2 (Proteintech, 10,412‐1‐AP), anti‐RAD51 (Abcam, ab176458), anti‐DMC1 (Santa Cruz, sc‐22,768), anti‐PLZF (Santa Cruz, sc‐22,839), anti‐cleaved PARP1 (CST, 9544), anti‐MVH (Abcam, ab13840), anti‐p‐p53(Ser15) (CST, 9284S), anti‐p‐H3(Ser10) (CST, 9701S), and anti‐BrdU (Abcam, ab6326). Anti‐MEIOB antibody was a kind gift from Mengcheng Luo (Wuhan University).

### 4.3 Histological analysis

Mouse testis and epididymis were fixed in Bouin's solution (Sigma) and embedded in paraffin after dehydration in a series of increasing concentrations of ethanol. After section, the slides were deparaffinised, stained with haematoxylin and eosin (H/E) and mounted. The images were captured by an upright microscope (Leica DM4000).

### 4.4 Cell culture and cell viability assay

Mouse ESCs were cultured in DMEM medium supplemented with 15% FBS, 1% penicillin and streptomycin, 1× non‐essential amino acids (NEAA), 1× l‐glutamine, 10 ng/mL LIF (Santa Cruz), and 0.1 mM β‐mercaptoethanol. Cells were grown in humidified incubator (37°C) with 5% CO_2_. For cell viability assay, ESCs were seeded in 6‐well plates (1 × 10^5^ cells per well) and 1 μM 4‐OHT was added to the cell culture medium. Cells were passaged and cell numbers were counted every 2 days.

### 4.5 Western blotting analysis

Proteins were extracted using NETN300 lysis buffer (300 mM NaCl, 0.5 mM EDTA, 0.5% NP‐40, 20 mM Tris–HCl pH 8.0). Cell lysates were incubated on ice for 10 min. After centrifuged at 12,000*g* for 10 min at 4°C, the supernatants were collected as protein extracts. The protein extracts were separated by SDS‐PAGE and then transferred onto PVDF membrane (Millipore). The membranes were blocked by 5% non‐fat milk in PBST buffer (PBS with 20% Triton X‐100), incubated with primary antibodies overnight at 4°C, washed with PBST, and incubated with HRP‐conjugated secondary antibodies (Jackson ImmunoResearch). Proteins of interest were detected by chemiluminescence system with ECL reagents.

### 4.6 Surface spread of spermatocytes

Meiotic chromosome spreads of spermatocytes were prepared as described previously.^9^ Briefly, testes were dissected from 3‐week‐old mice and digested by 1 mg/mL type IV collagenase for 30 min at 37°C. The cell pellets were resuspended in hypotonic buffer (30 mM Tris–HCl pH 8.2, 50 mM sucrose, 17 mM sodium citrate) and were kept for 10 min. After centrifugation, cells were resuspended in 100 mM sucrose for 5 min. The cell suspension was mixed with equal volume of fixation buffer (1% PFA, 0.15% Triton X‐100, 10 mM sodium borate, pH 9.2) and was spread onto slides and air dried.

### 4.7 IF staining

For IF staining of meiotic spreads, the slides were treated with 0.5% Triton X‐100 for 10 min and were incubated with primary antibodies for 3 h. After washing in 1× PBS for three times, the slides were incubated with Alexa Fluor 488 or 594‐conjugated secondary antibodies for 30 min. Finally, the slides were stained with Hoechst 33342 and mounted with anti‐fade reagents. The images were captured by an inverted fluorescence microscope (ECLIPSE Ti2‐E, Nikon).

For IF staining of frozen sections, the slides were incubated with primary antibodies overnight and secondary antibodies for 1 h, respectively. The images were taken by a laser scanning confocal microscope (STELLARIS 5, Leica Microsystems). The fluorescence intensity was quantified by ImageJ.

### 4.8 Separation of nuclear and cytoplasmic proteins

Cell pellets were treated with lysis buffer 1 (10 mM Tris–HCl pH 8.0, 10 mM KCl, 1.5 mM MgCl_2_, 1 mM EDTA) and were kept on ice for 20 min. 0.15% NP40 was added to the lysates and incubated for 2 min. After centrifugation, the supernatant was collected as cytoplasmic protein. The insoluble fraction was washed with ice‐cold 1× PBS for three times and treated with lysis buffer 2 (10 mM Tris–HCl pH 8.0, 1.5 mM MgCl_2_, 400 mM NaCl, 1 mM EDTA) for 15 min. After centrifugation, the supernatant was collected as nuclear protein.

### 4.9 Germ cell enrichment

Germ cell enrichment was performed as described previously.^9^ Mouse testes were isolated and digested in 1 mg/mL type IV collagenase for 10 min at 37°C. After centrifugation, cell pellets were further digested with trypsin for 10 min. The reaction was then stopped by adding foetal bovine serum and cells were then centrifugated at 400 *g* for 2 min. The cell pellets were resuspended in DMEM medium supplemented with 10% FBS and 1% penicillin and streptomycin, and then cultured in dishes at 37°C with 5% CO_2_. One hour later, the supernatant cells were collected for subsequent Western blotting analysis.

### 4.10 Statistical analysis

The statistical significance between two groups was evaluated using two‐tailed unpaired Student's *t*‐test.

## AUTHOR CONTRIBUTIONS

Lejun Li, Lin‐Yu Lu, and Yidan Liu designed research. Zhenghui Tang, Zhongyang Liang, Bin Zhang, and Xiaohui Xu performed research. Zhenghui Tang, Zhongyang Liang, Bin Zhang, Xiaohui Xu, Peng Li, and Lejun Li analysed data. Zhenghui Tang, Zhongyang Liang, Lin‐Yu Lu, and Yidan Liu wrote the article.

## FUNDING INFORMATION

This work is funded by Zhejiang Provincial Natural Science Foundation (LQ21C120001 and LZ21H040001) and National Natural Science Foundation of China (32070829, 32100676, and 32370900).

## CONFLICT OF INTEREST STATEMENT

The authors declare no conflict of interests.

## Data Availability

All data supporting the findings of this study are available within the paper.
